# *Legionella* Infection Risk from Domestic Hot Water

**DOI:** 10.3201/eid1003.020707

**Published:** 2004-03

**Authors:** Paola Borella, M. Teresa Montagna, Vincenzo Romano-Spica, Serena Stampi, Giovanna Stancanelli, Maria Triassi, Rachele Neglia, Isabella Marchesi, Guglielmina Fantuzzi, Daniela Tatò, Christian Napoli, Gianluigi Quaranta, Patrizia Laurenti, Erica Leoni, Giovanna De Luca, Cristina Ossi, Matteo Moro, Gabriella Ribera D’Alcalà

**Affiliations:** * University of Modena and Reggio E., Modena, Italy; † University of Bari, Bari, Italy; ‡ Catholic University in Rome, Rome, Italy; § University Institute of Physics Sciences, Rome, Italy,; ¶ University of Bologna, Bologna, Italy; # S. Raffaele Hospital, Milan, Italy; ** University Federico II of Naples, Naples, Italy

**Keywords:** Legionella spp., Pseudomonas spp., water microbial contamination, domestic hot water systems home colonization, Legionella multicentric survey, community legionellosis

## Abstract

We investigated *Legionella* and *Pseudomonas* contamination of hot water in a cross-sectional multicentric survey in Italy. Chemical parameters (hardness, free chlorine, and trace elements) were determined. *Legionella* spp. were detected in 33 (22.6%) and *Pseudomonas* spp. in 56 (38.4%) of 146 samples. Some factors associated with *Legionella* contamination were heater type, tank distance and capacity, water plant age, and mineral content. *Pseudomonas* presence was influenced by water source, hardness, free chlorine, and temperature. *Legionella* contamination was associated with a centralized heater, distance from the heater point >10 m, and a water plant >10 years old. Furthermore, zinc levels of <20 μg/L and copper levels of >50 μg/L appeared to be protective against *Legionella* colonization. *Legionella* species and serogroups were differently distributed according to heater type, water temperature, and free chlorine, suggesting that *Legionella* strains may have a different sensibility and resistance to environmental factors and different ecologic niches.

Legionnaires’ disease is normally acquired by inhalation or aspiration of legionellae from a contaminated environmental source. The first evidence of the association between potable water from shower and nosocomial legionellosis was reported approximately 20 years ago (*1*), and the hot water system is thought to be the most frequent source of cases or outbreaks within a hospital (*2*,*3*), where patients may be at a higher risk for a severe infection (*4*–*6*). Relatively little is known about sporadically occurring cases of community-acquired legionellosis, which accounts for most infections (*7*,*8*), although correlation analyses suggest that a substantial proportion of these cases may be residentially acquired and associated with bacteria in hot water distribution systems (*9*).

*Legionella* spp. have been isolated from water with a temperature as high as 63°C, and the contamination is associated with other bacteria and protozoa (*10*,*11*). Biofilm formation can provide a means for survival and dissemination of *L. pneumophila* (*12*,*13*), interfering with efforts to eradicate bacteria from water systems (*14*,*15*). The accumulation of microorganisms on the pipeline surfaces and the formation of biofilms are influenced by many factors, such as surface materials, concentration and quality of nutrients and disinfectants, temperature and hydraulics of the system, and pipe surface roughness (*16*).

To assess the potential public health impact of *Legionella* colonization at a domestic level, a descriptive multicentric study was undertaken to identify and quantify the levels of the microorganism in a substantial number of Italian domestic hot water samples. *Pseudomonas* spp. are part of the natural population of the water, but some species should be considered as opportunistic pathogens. Furthermore, *Pseudomonas* may compete with *Legionella* to grow in the aquatic environment (*17*,*18*); thus we also evaluated *Pseudomonas* colonization.

We addressed three specific aims: 1) to estimate the frequency of *Legionella* colonization and severity of contamination at the domestic level; 2) to identify potential risk factors for contamination relative to distribution systems and water characteristics; 3) and to define the relative role of each risk factor and suggest possible remediation. Lastly, risk for legionellosis was retrospectively evaluated by collecting information about pneumonia symptoms recorded by residents at home.

## Methods

### Sample Collection

From May through June 2002, a total of 146 water samples were collected from private homes of six towns (Milan, Modena, Bologna, Rome, Naples, Bari) representative of different Italian regions (Northern, Central, and Southern Italy). A similar number of samples were taken from each town; selection was made on the basis of the water distribution systems inside the town and building and heater types in each area. After we identified each building, we asked a random family (in case of a condominium) to participate in the study, i.e., to complete our questionnaire and give informed consensus for water collection. Laboratory examinations were free, and at the end of the study each participating family received a letter with results of *Legionella* analysis.

Hot water samples were drawn from the bathroom outlets (shower heads or bathroom tap) in three sterile 1-L glass bottles after a brief flow time (to eliminate cold water inside the tap or flexible shower pipe). To neutralize residual free chlorine, sodium thiosulphate was added in sterile bottles for bacteriologic analysis, whereas acid-preserved glass bottles were used for chemical determinations. Collection bottles were returned to the laboratory immediately after sampling for bacteriologic and chemical-physical examination; if analyses would not begin within 24 hours, samples were kept at >4°C and processed within 48 hours of collection.

### Microbiologic Analysis

To detect *Legionella* spp., 2-L water samples were concentrated by membrane filtration (0.2-μm-pore–sized polyamide filter, Millipore, Billerica, Massachusetts, USA). The filter membrane was resuspended in 10 mL of original sample water and vortex-mixed for 10 min. To reduce contamination by other microorganisms, 5 mL of this suspension was heat-treated (50°C for 30 min in a water bath) (*19*). Two aliquots of 0.1 mL of the original and concentrated specimens (heat-treated and untreated, 1:10 diluted and undiluted) were each spread on duplicate plates of modified Wadowsky-Yee selective medium (Oxoid Ltd., Basingstoke, Hampshire, UK). The plates were incubated at 36°C in a humidified environment with at least 2.5% CO_2_ for 10 days and read from day 5 at the dissecting microscope. Suspected colonies with a mottled surface or an iridescent and faceted cut-glass appearance, were counted from each sampling. All colonies from plates with <10 and 10–20 random colonies were subcultured on buffered charcoal yeast extract (BCYE) agar (with cysteine) and charcoal yeast extract agar (cysteine-free) media (Oxoid) for >2 days. Only colonies grown on BCYE were subsequently identified by an agglutination test (*Legionella* Latex Test, Oxoid). The test allows a separate identification of *L. pneumophila* serogroup 1 and serogroups 2–14 and detection of seven *Legionella* (polyvalent) species (other than *L. pneumophila*), which have been implicated in human disease: *L. longbeachae*, *L. bozemanii* 1 and 2, *L. dumoffii*, *L. gormanii*, *L. jordanis*, *L. micdadei*, *L. anisa*. *Legionella*-like bacteria serologically nonidentifiable (in total 4 colonies) are excluded from the account, awaiting a different confirmation by DNA sequence (polymerase chain reaction [PCR]-method). The results are expressed as CFU/L and the detection limit of the procedure was 25 CFU/L (mean value of two plates). All the research units participated in a quality control for *Legionella* detection in water that was organized by the National Health Institute, through a periodic distribution of water samples added with unknown *Legionella* species and concentration. The total microbial counts at 36°C and 22°C were obtained twice by the pour-plate method on plate count agar (Oxoid). The plates were incubated at 36°C for 48 h or at 22°C for 72 h.

To isolate *Pseudomonas* spp., 100-mL and 10-mL water samples were filtered through a 0.45-μm-pore–size membrane (Millipore). If the number of bacteria was high, suitable dilutions were made. The membranes were placed on *Pseudomonas* cetrimide fucidin cephalosporin (CFC) agar (Oxoid) and incubated at 30°C for 48 h. Each type of oxidase-positive colony was counted.

### Physical and Chemical Analyses

Water temperature and residual free chlorine (DPD method, colorimetric) were determined at the time of sample collection. Standard techniques were used to measure oxidizability and water hardness. Concentrations of calcium, magnesium, iron, manganese, copper, and zinc were measured by flame atomic absorption spectrophotometer (Perkin-Elmer, Wellesley, MA, mod 5000) on acidified samples (1% HNO_3_) concentrated by boiling.

### Risk Factors

A detailed standardized questionnaire was developed to evaluate risk factors possibly associated with colonization. The first part collected information on family characteristics (number of components, age and sex, length of stay in residence) and on pneumonia events during their stay in the home. The second part was devoted to home data: type (flat, single house, villa), flats in the building, home floor, home rooms and bathrooms, building age, type of water supply, and disinfection systems used. The third part collected information on the heating system (central or independent, electric or gas heater), distance of the sample site from the water distribution point, existence of a tank and its volume, age of the system, service frequency, and existence and characteristics of a softening and water recycling systems. Water operating temperature (temperature at the distribution site) was also recorded.

### Statistical Analysis

All statistical calculations were made with SPSS/pc (SPSS Inc, Chicago, IL). Logarithmic transformations were used in statistical analyses to normalize the nonnormal distributions, and results are presented as geometric means. The bacteriologic data were converted into log_10_ (x+1). When possible, variables were categorized into dichotomous ones. The results were analyzed by correlation analysis, t test, one-way analysis of variance (ANOVA), and by chi-square test. Odds ratios (OR) and 95% confidence intervals (CI) were calculated to assess categorical risk variables associated with microbial contamination. Variables that were significant in the univariate analysis were entered in a multiple logistic regression model. By using conditional logistic regression models, independent predictors of colonization were established. Variables were retained in the model if the likelihood ratio test was significant (p < 0.05).

## Results

### Descriptive Data

Table 1 shows general characteristics of the examined water in terms of supply and distribution systems. Five heating systems were recognized, corresponding to those more frequently used at the domestic level, although with geographic differences, as the centralized systems were mainly adopted in northern Italy.

**Table 1:** Characteristics of water supply and distribution systems in the examined buildings (N = 146)

Characteristic	Frequency no. (%)
Type of water	
Groundwater	67 (46.2)
Mixture water	79 (53.8)
Disinfection	
Cl dioxide	58 (39.7)
Na hypochlorite	30 (20.6)
Cl dioxide + Na hypochlorite	32 (22.2)
None	26 (17.5)
Plumbing material	
Metal	131 (89.7)
Plastic	15 (10.3)
Type of heater	
Independent	
Gas, tank	30 (20.5)
Electric	22 (15.1)
Gas, instant	55 (37.7)
Central	
House	36 (24.7)
Neighborhood	3 (2.1)
Softening system	
Absent	124 (84.9)
Present	22 (15.1)
Hot water circulation	
Absent	117 (80.1)
Present	29 (19.9)
Plant maintenance	
Never/every 2 years	26 (17.8)
Once/year	100 (68.5)
Every 6 months	20 (13.7)

Table 2 shows chemical and microbiologic qualities of hot water samples. When samples were grouped according to their origin (mixture or groundwater), groundwater had significantly higher levels of calcium (105.7 ± 32.1 mg/L vs. 68.8 ± 31.3 mg/L, p < 0.001) and magnesium (21.5 ± 16.0 mg/L vs. 14.9 ± 6.0 mg/L, p < 0.01) and was harder (35.4 ± 13.0 vs. 22.1 ± 8.8°F, p < 0.001). *Pseudomonas* spp. were isolated from 56 of 146 (38.4%) samples, with levels ranging from 1 to 6.4 x 10^4^ CFU/100 mL; 85.7% of positive samples contained fewer than 10^3^ CFU/100 mL.

**Table 2:** Chemical, physical, and microbiologic characteristics of the examined hot water samples

Characteristic	Mean	SD	Minimum	Maximum	
Ca (mg/L)	85.0	33.0	0.1	252.0	
Mg (mg/L)	17.6	10.6	0.1	90.1	
Total hardness (°F)	28.3	11.4	0.3	100.1	
Sampling temperature (°C)	41.9	12.4	17.0	65.0	
Operating temperature (°C)	52.9	10.0	20.0	90.0	
	Geometric mean	Median	5th percentile	95th percentile	
Fe (μg/L)	15.0	21.0	0.1	171.5	
Mn (μg/L)	2.4	3.0	0.1	13.9	
Cu (μg/L)	11.5	14.2	0.1	87.5	
Zn (μg/L)	62.6	100.0	1	776.4	
Free Cl (μg/L)	9.2	26.4	0.0	250.0	
Oxidizability (mg/L O_2_)	0.63	0.64	0.24	1.70	
*Pseudomonas* spp. (CFU/100 mL)^a^	139.2	223.6	1.0	1.0 × 10^4^	
Total count at 36°C (CFU/mL)^a^	98.6	95.0	3.4	1.7 × 10^3^	
Total count at 22°C (CFU/mL)^a^	50.4	30.0	4.0	3.9 × 10^3^	

^a^Positive samples only.

A total of 33 (22.6%) samples of 146 were contaminated by *Legionella* spp., and *L. pneumophila* (Table 3) was the most frequently isolated species (75.8% of isolates). In the positive samples, the mean number of legionellae was 1.17 x 10^3^ CFU/L (range 25 to 8.7 x 10^4^ CFU/L); three samples (9.2%) contained >10^4^ CFU/L, none of which were *L. pneumophila* serogroup 1. Although we examined colonies with different morphologic traits, the agglutination test did not reveal multiple species or serotypes in a single water sample.

**Table 3:** Characteristics of *Legionella* contamination in the examined domestic hot water

Characteristic	*Legionella* spp. total	*L. pneumophila* serogroup 1	*L. pneumophila* serogroups 2–14	Other *Legionella* species
Positive samples n (%)	33/146 (22.6)	6/33 (18.2)	19/33 (57.6)	8/33 (24.2)
Count (CFU/L)				
Geometric mean	1.17 × 10^3^	0.96 × 10^3^	0.94 × 10^3^	2.30 × 10^3^
Median	1.85 × 10^3^	0.89 × 10^3^	1.85 × 10^3^	3.16 × 10^3^
5th percentile	54	100	25	100
95th percentile	4.1 × 10^4^	5 × 10^3^	3 × 10^4^	8.8 × 10^4^
Distribution n (%)				
1–9.9×10^2^ CFU/L	15/33 (45.4)	3/6 (50.0)	9/19 (47.4)	3/8 (37.5)
10^3^–9.9 × 10^3^ CFU/L	15/33 (45.4)	3/6 (50.0)	8/19 (42.1)	4/8 (50.0)
>10^4^ CFU/L	3/33 (9.2)	0/6 (0.0)	2/19 (10.5)	1/8 (12.5)

### Univariate Examination of Risk Factors

The risk for microbial contamination according to the system characteristics was evaluated by applying a univariate logistic regression (Table 4). A central warm water system and distance of the water from the heating point >10 m were strongly associated with the risk for *Legionella* contamination (OR 9.24 and 8.10, respectively, p < 0.001). Other significant and positive associations were observed with tank volume, plant age, flooring in the home, and total number of flats in the building. *Pseudomonas* contamination was positively associated with heating system age and negatively with tank distance and water operating temperature. *Pseudomonas* was also associated with the particular water source, with 65.3% of groundwater colonized versus 12.3% of mixture water (OR 13.44; 95% CI 5.02 to 36.03, p < 0.001).

**Table 4:** Univariate analysis of system and building characteristics associated with microbial contamination^a^

Characteristic	OR (95% CI)			
	*Legionella* spp. yes/no (33/113)	*Pseudomonas* spp. yes/no (56/90)	Total count at 36°C high/low (62/84)^b^	Total count at 22°C high/low (35/111)^b^
Central heater	9.24^c^ (3.87 to 22.05)	0.87 (0.40 to 1.85)	0.92 (0.44 to 1.94)	1.36 (0.59 to 3.12)
Distribution site distance >10 m	8.10^c^ (3.41 to 19.23)	0.47* (0.22 to 1.00)	1.08 (0.54 to 2.17)	1.51 (0.69 to 3.32)
Tank capacity >100 L	5.21^c^ (2.14 to 12.67)	0.58 (0.24 to 1.43)	0.58 (0.24 to 1.39)	0.64 (0.22 to 1.83)
Age of heating plant >10 y	3.24^d^ (1.38 to 7.59)	1.85^e^ (0.94 to 3.64)	1.14 (0.59 to 2.19)	1.58 (0.73 to 3.43)
House floor >3rd	2.35^e^ (1.04 to 5.30)	1.38 (0.66 to 2.87)	0.94 (0.45 to 1.96)	1.24 (0.54 to 2.83)
Apartments >12/building	2.26^e^ (0.99 to 5.18)	1.48 (0.75 to 2.91)	1.00 (0.52 to 1.94)	1.13 (0.53 to 2.44)
Sampling temperature >50°C	0.62 (0.26 to 1.49)	0.91 (0.24 to 3.40)	1.16 (0.54 to 2.51)	1.22 (0.54 to 2.75)
Operating temperature >50°C	0.69 (0.29 to 1.67)	0.32^d^ (0.15 to 0.70)	1.74 (0.80 to 3.76)	0.57 (0.24 to 1.36)

^a^OR, odds ratio; CI, confidence interval. ^b^High >100 CFU/mL. ^c^p < 0.001. ^d^p < 0.01. ^e^p < 0.05.

The univariate regression was then applied to study the association between microbiologic data and water chemical parameters (Table 5). Seven factors were independently protective against *Legionella* colonization: high levels of copper, hardness, oxidizability, and free chlorine and low concentrations of zinc, iron, and manganese. Lower levels of zinc and manganese were also associated with lower total count at 36°C and 22°C. *Pseudomonas* was positively associated with total hardness and iron <20 μg/L, whereas residual free chlorine significantly inhibited *Pseudomonas*. When water samples were grouped according to their trace element levels, water samples (n = 12) characterized by concentrations of zinc <100 μg/L, iron <20 μg/L, and copper >50 μg/L were all negative for *Legionella*. No other system or water parameters were associated with bacterial contamination of the examined samples.

**Table 5:** Univariate analysis of water chemical parameters associated with microbiologic data^a^

Chemical	*Legionella* spp. (yes/no)	*Pseudomonas* spp. (yes/no)	Total count at 36°C (high/low)^b^	Total count at 22°C (high/low)^b^	
	OR (95% CI)	OR (95% CI)	OR (95% CI)	OR (95% CI)	
Cu ≥50 μg/L	0.14^c^ (0.02 to 1.13)	0.99 (0.38 to 2.56)	0.64 (0.24 to 1.68)	0.71 (0.22 to 2.28)	
Zn <100 μg/L	0.33^d^ (0.14 to 0.76)	1,21 (0.62 to 2.35)	0.38^d^ (0.19 to 0.75)	0.29^d^ (0.13 to 0.67)	
Fe <20 μg/L	0.37^c^ (0.16 to 0.85)	1.96^c^ (1.00 to 3.86)	050^c^ (0.25 to 0.97)	0.74 (0.34 to 1.58)	
Mn <3 μg/L	0.42^c^ (0.19 to 0.94)	1.50 (0.76 to 2.94)	0.23^e^ (0.11 to 0.45)	0.31^d^ (0.14 to 0.70)	
Hardness >25°F	0.41^c^ (0.18 to 0.89)	3.91^e^ (1.80 to 8.52)	1.92(0.96 to 3.86)	1.03 (0.47 to 2.26)	
Oxidizability >0.5 mg/L O_2_	0.42^c^ (0.18 to 0.95)	1.12 (0.52 to 2.42)	1.01 (0.49 to 2.07)	0.29^d^ (0.12 to 0.68)	
Free Cl present	0.51 (0.23 to 1.15 )	0.35^d^ (0.17 to 0.73)	1.52 (0.73 to 3.13)	0.49 (0.22 to 1.09)	

^a^OR, odds ratio; CI, confidence interval. ^b^High >100 CFU/mL. ^c^p < 0.05. ^d^p < 0.01. ^e^p < 0.001.

### Multivariate Examination of Risk Factors

The data were reanalyzed by means of multivariate conditional logistic regression models. A central heating system, distance from heating point >10 m, and a system >10 years old were each independently associated with higher risk of *Legionella* colonization, whereas water with levels of copper >50 μg/L and zinc <100 μg/L were predictive of no contamination (Table 6). For *Pseudomonas*, only groundwater remained highly predictive of colonization (OR 12.69; 95% CI 2.66 to 44.00, p < 0.001), whereas an operating temperature >50°C was predictive of noncontaminated samples (OR 0.25; 95% CI 0.11 to 0.98, p < 0.05).

**Table 6:** Multiple logistic regression of system and water characteristics associated with *Legionella* contamination^a^

Variable	*Legionella* spp. yes/no
	OR (95% CI)
Central heater	4.88 (1.61 to 14.76)^b^
Distribution site distance >10 m	4.55 (1.55 to 13.33)^c^
Zn <100 μg/L	0.22 (0.07 to 0.66)^c^
Heating system age >10 yr	3.68 (1.25 to 10.82)^d^
Cu >50 μg/L	0.08 (0.01 to 0.97)^d^

^a^OR, odds ratio; CI, confidence interval. ^b^p < 0.005. ^c^p < 0.01. ^d^p < 0.05.

### Risk Factors and *Legionella* Species

The percentage distribution of *Legionella* species differed significantly according to the heater system (chi-square = 14.00, p < 0.05). Electric heaters were legionellae-free; the gas-heated independent systems had little contamination (10.0% of those with tank and 16.4% of those without) and were mainly colonized by either *L. pneumophila* serogroup 1 or non-*pneumophila Legionella* species. In the centralized heating systems of both single buildings and neighborhoods, *Legionella* colonization was higher (52.8% and 66.7%, respectively), and *L. pneumophila* serogroups 2–14 were the most frequently isolated serotypes. Germ concentration did not differ according to the heater type.

Table 7 shows that water temperature, level of free chlorine, and *Pseudomonas* contamination differed according to *Legionella* species (Table 7). Water samples contaminated by *L. pneumophila* serogroup 1 were characterized by significantly lower operating temperature compared to that of the other groups, whereas water samples positive for *L. pneumophila* serogroups 2–14 had lower residual chlorine and higher *Pseudomonas* count.

**Table 7:** Differences in some water parameters according to *Legionella* species

Characteristic	*L. pneumophila* serogroup 1 (n = 6) Mean ± SD	*L. pneumophila* serogroups 2–14 (n = 19) Mean ± SD	*Legionella* other species (n = 8) Mean ± SD	F score (p)
Sampling temperature (°C)	29.4 ± 11.8	48.9 ± 4.4^a^	31.2 ± 15.5	15.17 (<0.001)
Operating temperature (°C)	43.3 ± 12.1^a^	53.7 ± 5.9	57.0 ± 4.4	6.91 (<0.005)
	Geometric mean	Geometric mean	Geometric mean	
Free chlorine (μg/L)	78.0	2.6^a^	32.3	7.86 (<0.002)
*Pseudomonas* spp. (CFU/100 mL)	0.4	19.1^b^	0.6	3.31 (<0.05)

^a^p < 0.05 versus the other two groups. ^b^p < 0.05 versus *Legionella* other species.

### Risk Assessment

The reported frequency of pneumonia symptoms was double among persons living in the legionellae-positive homes compared to those living in legionellae-free buildings (8 cases in 95 residents vs. 15 cases of 333 residents), but the difference was not significant (OR 1.95; 95% CI 0.80 to 4.75). Results did not change by correcting for the duration of residence of each person in the examined house.

## Discussion

In our study, *Legionella* spp. were isolated in 22.6% of domestic hot water samples, with a mean number of legionellae in positive samples of 1.17 x 10^3^ CFU/L (geometric mean); the highest concentration was 8.7 x 10^4^. In previous studies in Finland and Germany, the occurrence of legionellae was similar (30% and 26%, respectively) as well as the contaminating concentration (*20*,*21*). In an Italian study of hot water samples taken from swimming pool showers, 27% were positive for *Legionella* spp. and 46% for *P. aeruginosa* (*18*), findings in line with results of our study on domestic water plants. According to a survey in Germany (*22*), *L. pneumophila* is by far the most abundant species in potable and environmental water samples, as >75% of positive samples were contaminated by *L. pneumophila*.

We could not verify seasonal variability in the contamination, because all samples were taken in the spring. Recent studies, however, found that contamination was consistent throughout the year, both in terms of the species of legionellae isolated and in the concentration of organisms (*18*), suggesting that the occurrence of Legionnaires’ disease most frequently in the summer is not necessarily linked to a higher water contamination.

By comparing the environmental factors associated with *Legionella* and *Pseudomonas* occurrence, substantial differences in the microbes’ sensitivity to these factors were observed. *Pseudomonas* was not influenced by system characteristics but strongly affected by water parameters. Thus, free chlorine and operating temperature appeared to inhibit these microbes, whereas groundwater origin, which influences higher degree of hardness, was found to favor *Pseudomonas* occurrence. The negative effect of chlorine and the positive influence of hardness, particularly higher calcium level, have been already observed in other studies on *Pseudomonas* water contamination (*17*,*23*).

Conversely, system and building characteristics were the main predictors for *Legionella* in domestic hot water. Thus, residing at higher floors of large buildings with many apartments and with older, centralized water heating systems increased the risk for *Legionella* contamination compared to living in apartments with independent water heater systems and a short distance from the sampling point to the hot water distribution site. Among independent heaters, electric ones appeared to be most protective against contamination, whereas the opposite was observed in previous studies in Quebec City, where temperature of electric heaters was significantly lower than that of fossil-fuel heaters (*24*). In a representative sample of Wellington, New Zealand, domestic residences with electrically heated hot water systems, no *Legionella* spp. were isolated by culture, but PCR tests were positive for *Legionella* in 12 homes, some with hot water temperatures >60°C, suggesting that the bacteria are killed during passage through the hot water tank (11).

In our study, *Legionella* presence was not affected by the origin of water (groundwater vs. mixture), pipe materials, water temperature, or concentration of chlorine, and the negative association of *Legionella* with hardness and oxidizability disappeared in the multilogistic regression analysis. When the potable system was adopted, *Legionella* was found in both chlorinated and untreated water, confirming the low efficacy of this disinfecting system on microbe eradication (*25*). In addition, bacteria from a chlorinated water system may be more resistant to combined and free chlorine than bacteria from unchlorinated systems (*26*).

The examined domestic water samples were not colonized by multiple serotypes or strains, a common finding in hospitals, hotels, and spas (*27*–*29*). This result could depend on different distribution systems and frequency of water use between private and public buildings.

Because the contaminating organism (*L. pneumophila* serogroup 1, *L*. *pneumophila* serogroups 2–14, or non-*pneumophila Legionella* spp.) was specific to a system, we could examine differences in distribution of species according to the system and water characteristics. These differences have been insufficiently evaluated in previous studies, but recent studies demonstrated that intracellular replication, cytopathogenicity, and infectivity to mammalian and protozoan cells also vary with *Legionella* species (*30*,*31*).

Our hypothesis is that *Legionella* strains substantially differ in their sensitivity to environmental risk factors and, as a consequence, may have different ecologic niches. *L. pneumophila* serogroup 1, responsible for approximately 80%–90% of Legionnaires’ disease cases (*32*), was predominantly isolated from independent water heating systems, despite the fact that they were less frequently contaminated. Furthermore, compared with the other legionellae, serogroup 1 was found in water with a lower temperature, less *Pseudomonas* contamination, and a relatively higher residual chlorine concentration. Taking results together, *L. pneumophila* serogroup 1 appears to survive and grow in systems with a short distance between the hot water distribution site and the distal outlets. In agreement with our findings, a recent study on contaminated dental units recovered *L. pneumophila* serogroup 1 in nearly all sites positive for *Legionella* species (*33*). In these conditions, the possibility of contaminated aerosol inhalation might be more frequent for *L. pneumophila* serogroup 1, despite the fact that this serogroup is not the most frequently isolated in hot water systems. If our hypothesis is correct, most probably simple hygienic procedures, like good cleaning practice and periodically replacing shower heads, would be effective in reducing the number of infections. From our experience with epidemic clusters of nosocomial legionellosis in a hospital mainly contaminated by *L. pneumophila* serogroups 2–14 with rare isolates of *L. pneumophila* serogroup 1, we observed that introducing adequate cleaning procedures in the bathroom and surveillance by health personnel was sufficient to avoid further cases, even when the central hot water distribution systems were not decontaminated (*34*).

Our findings show the possible effect of trace elements on *Legionella* in hot water samples. Experimental studies have shown that *Legionella* spp. are affected by osmolarity (*35*) and metal concentration (*36*) and that iron limitation in vitro reduces bacteria growth and expression of the zinc-metalloprotease that is an important pathogenicity factor (*37*). We show that hot water samples low in iron, zinc, and manganese, but rich in copper, predicted the absence of *Legionella* colonization, confirming their roles as growth promoters or inhibitors.

Of particular interest is the inverse relationship between copper levels and *Legionella* presence. In the examined water, the risk of *Legionella* contamination was approximately six times lower when copper levels exceeded 50 μg/L, without influencing *Pseudomonas* contamination. In other studies, copper concentrations low enough to be commonly found in drinking water reduced numbers of coliform bacteria (*13*). Thus, we emphasize that this trace element influences some, but not all, bacterial growth (*33*). To control *Legionella* in hot water systems, methods that release copper and silver ions electrolytically in water may represent a promising solution (*38*–*41*). Although both metals play a role in limiting bacterial colonization, copper seems to better penetrate biofilm. Amoebae, the natural hosts of legionellae, have not been controlled successfully in vitro by adding metal (*42*), suggesting that legionellae survive inside protozoa and are destroyed by metal ions when released into free water.

The risk of getting pneumonia was 1.95 higher among residents in the legionellae-positive homes than in residents of the legionellae-negative buildings, but the difference was not significant and was similar to that found in previous studies (*19*). Legionellae concentrations of 3–7,000 CFU/L could be sufficient to produce one case per year in a susceptible population (*43*), and these contamination levels correspond to those found in our study at the domestic level. In a recent epidemiologic survey on seropositivity in residents of homes with and without *Legionella* in the water systems, the prevalence of anti-*Legionella* antibodies was twice as high in persons in homes with legionellae as in those persons whose homes did not have legionellae (*44*). The antibodies were most likely the result of asymptomatic infections caused by exposure in their home water supply, as no cases of pneumonia in the exposed population were reported. Most cases of sporadic legionellosis are not reported to health authorities in Italy as well as in other countries, and finding an association with a specific source of infection such as domestic contamination is rare (*45*).

Our observations suggest that *Legionella* species should be considered when examining environmental contamination, which is essential to better evaluate environmental risk factors and select the most appropriate prevention and control measures (*46*). To limit *Legionella* colonization at the domestic level, we suggest simple and general measures: 1) use independent domestic water heaters, 2) maintain high cleaning standards, 3) periodically replace components of the system which could favor presence or dissemination of bacteria, and 4) have a water copper content >50 μg/L. We do not believe disinfecting measures at the domestic level are needed, considering that our retrospective study on pneumonia in residents did not show a relevant evidence of risk in colonized buildings.

## References

[1] Tobin JO, Beare J, Dunnill MS, Fisher-Hoch S, French M, Mitchell RG, Legionnaires’ disease in a transplant unit: isolation of the causative agent from shower baths. Lancet. 1980;2:118–21. 10.1016/S0140-6736(80)90005-7 6105294

[2] Sabria M, Yu VL. Hospital-acquired legionellosis: solutions for a preventable infection. Lancet Infect Dis. 2002;2:368–73. 10.1016/S1473-3099(02)00291-8 12144900

[3] Anaissie EJ, Penzak-Scott R, Dignani MC. The hospital water supply as a source of nosocomial infections: a plea for action. Arch Intern Med. 2002;162:1483–92. 10.1001/archinte.162.13.1483 12090885

[4] Pedro-Botet ML, Sabria-Leal M, Sopena N, Manterola JM, Morera J, Blavia R, Role of immunosuppression in the evolution of Legionnaires’ disease. Clin Infect Dis. 1998;26:14–9. 10.1086/516258 9455504

[5] Yu VL. Resolving the controversy on environmental cultures for *Legionella* a modest proposal. Infect Control Hosp Epidemiol. 1998;19:893–7. 10.1086/647759 9872524

[6] Tkatch LS, Kusne S, Irish WD, Krystofiak S, Wing E. Epidemiology of *Legionella pneumonia* and factors associated with *Legionella*-related mortality at a tertiary care center. Clin Infect Dis. 1998;27:1479–86. 10.1086/515040 9868664

[7] Atlas RM. *Legionella*: from environmental habitats to disease pathology, detection and control. Environ Microbiol. 1999;1:283–93. 10.1046/j.1462-2920.1999.00046.x 11207747

[8] PHLS Communicable Disease Surveillance Centre. Legionnaires’ disease in Europe, 1997. Wkly Epidemiol Rec. 1998;73:257–61. 9763931

[9] Straus WL, Plouffe JF, File TM, Lipman HB, Hackman BH, Risk factors for domestic acquisition of legionnaires disease. Arch Intern Med. 1996;156:1685–92. 10.1001/archinte.156.15.1685 8694667

[10] Rogers J, Dowsett AB, Dennis PJ, Lee JV, Keevil CW. Influence of temperature and plumbing material selection on biofilm formation and growth of *Legionella pneumophila* in a model potable water system containing complex microbial flora. Appl Environ Microbiol. 1994;60:1585–92. 8017938 10.1128/aem.60.5.1585-1592.1994 PMC201521

[11] Bates MN, Maas E, Martin T, Harte D, Grubner M, Margolin T. Investigation of the prevalence of *Legionella* species in domestic hot water systems. N Z Med J. 2000;113:218–20. 10909936

[12] Murga R, Forster TS, Brown E, Pruckler JM, Fields BS, Donlan RM. Role of biofilms in the survival of *Legionella pneumophila* in a model potable-water system. Microbiology. 2001;147:3121–6. 11700362 10.1099/00221287-147-11-3121

[13] Zacheus OM, Lehtola MJ, Korhonen LK, Martikainen PJ. Soft deposits, the key site for microbial growth in drinking water distribution networks. Water Res. 2001;35:1757–65. 10.1016/S0043-1354(00)00431-0 11329678

[14] Lin YS, Stout JE, Yu VL, Vidic RD. Disinfection of water distribution systems for *Legionella.* Semin Respir Infect. 1998;13:147–59. 9643393

[15] Momba MNB, Kfir R, Venter SN, Cloete TE. An overview of biofilm formation in distribution systems and its impact on the deterioration of water quality. Water SA [serial on the Internet] 2000 Jan. Available from: http://www.wrc.org.za/publications/watersa/2000/January/jan00_p59.pdf

[16] Zacheus OM, Martikainen PJ. Effect of heat flushing on the concentrations of *Legionella pneumophila* and other heterotrophic microbes in hot water systems of apartment buildings. Can J Microbiol. 1996;42:811–8. 10.1139/m96-102 8776852

[17] Ribas F, Perramon J, Terradillos A, Frias J, Lucena F. The *Pseudomonas* group as an indicator of potential regrowth in water distribution systems. J Appl Microbiol. 2000;88:704–10. 10.1046/j.1365-2672.2000.01021.x 10792530

[18] Leoni E, Legnani PP, Bucci Sabattini MA, Righi F. Prevalence of *Legionella* spp. in swimming pool environment. Water Res. 2001;35:3749–53. 10.1016/S0043-1354(01)00075-6 11561639

[19] De Luca G, Stampi S, Lezzi L, Zanetti F. Effect of heat and acid decontamination treatments on the recovery of *Legionella pneumophila* from drinking water using two selective media. Microbiologica. 1999;22:203–8. 10423738

[20] Zacheus OM, Martikainen PJ. Occurrence of legionellae in hot water distribution systems of Finnish apartment buildings. Can J Microbiol. 1994;40:993–9. 10.1139/m94-159 7704835

[21] Zietz B, Wiese J, Brengelmann F, Dunkelberg H. Presence of *Legionellaceae* in warm water supplies and typing of strains by polymerase chain reaction. Epidemiol Infect. 2001;126:147–52. 11293675 PMC2869666

[22] Wellinghausen N, Frost C, Marre R. Detection of legionellae in hospital water samples by quantitative real-time LightCycler PCR. Appl Environ Microbiol. 2001;67:3985–93. 10.1128/AEM.67.9.3985-3993.2001 11525995 PMC93119

[23] Stobberingh EE, Drent M. Hardness of tap water and *Pseudomonas aeruginosa.* Neth J Med. 1996;49:98–9. 10.1016/0300-2977(96)00034-4 8824113

[24] Alary M, Joly JR. Risk factors for contamination of domestic hot water systems by legionellae. Appl Environ Microbiol. 1991;57:2360–7. 1768104 10.1128/aem.57.8.2360-2367.1991 PMC183576

[25] Borella P, Bargellini A, Pergolizzi S, Aggazzotti G, Curti C, Nizzero P, Prevention and control of *Legionella* infection in the hospital environment. Ann Ig. 2000;12:287–96. 11140095

[26] Ridgway HF, Olson BH. Chlorine resistance patterns of bacteria from two drinking water distribution system. Appl Environ Microbiol. 1982;44:972–87. 7149722 10.1128/aem.44.4.972-987.1982 PMC242125

[27] Rocha G, Verissimo A, Bowker R, Bornstein N, Da Costa MS. Relationship between *Legionella* spp. and antibody titres at a therapeutic thermal spa in Portugal. Epidemiol Infect. 1995;115:79–88. 10.1017/S0950268800058143 7641840 PMC2271569

[28] Darelid J, Hallander H, Lofgren S, Malmvall BE, Olinder-Nielsen AM. Community spread of *Legionella pneumophila* serogroup 1 in temporal relation to a nosocomial outbreak. Scand J Infect Dis. 2001;33:194–9. 10.1080/00365540151060824 11303809

[29] Borella P, Montagna MT, Romano-Spica V, Stampi S, Stancanelli G, Triassi M, Environmental diffusion of *Legionella* spp. and legionellosis frequency among patients with pneumonia: preliminary results of a multicentric Italian survey. Ann Ig. 2003;15:493–503. 14969302

[30] Gao LY, Susa M, Ticac B, Abu-Kwaik Y. Heterogeneity in intracellular replication and cytopathogenicity of *Legionella pneumophila* and *Legionella micdadei* in mammalian and protozoan cells. Microb Pathog. 1999;27:273–87. 10.1006/mpat.1999.0308 10545255

[31] Steinert M, Hentschel U, Hacker J. *Legionella pneumophila*: an aquatic microbe goes astray. FEMS Microbiol Rev. 2002;26:149–62. 10.1111/j.1574-6976.2002.tb00607.x 12069880

[32] Yu VL, Plouffe JF, Castellani-Pastoris M, Stout JE, Schousboe M, Distribution of *Legionella* species and serogroups isolated by culture in patients with sporadic community-acquired legionellosis: an international collaborative survey. J Infect Dis. 2002;186:127–8. 10.1086/341087 12089674

[33] Zanetti F, Stampi S, De Luca G, Fateh-Moghadam P, Bucci-Sabbatini MA, Cecchi L. Water characteristics associated with the occurrence of *Legionella pneumophila* in dental units. Eur J Oral Sci. 2000;108:22–8. 10.1034/j.1600-0722.2000.00763.x 10706473

[34] Borella P, Bargellini A, Pergolizzi S, Aggazzotti G, Curti C, Nizzero P, Surveillance of legionellosis within a hospital in northern Italy: May 1998 to September 1999. Eurosurveillance. 1999;4:118–20. 12631884 10.2807/esm.04.11.00061-en

[35] Heuner K, Brand BC, Hacker J. The expression of the flagellum of *Legionella pneumophila* is modulated by different environmental factors. FEMS Microbiol Lett. 1999;175:69–77. 10.1111/j.1574-6968.1999.tb13603.x 10361710

[36] Goldoni P, Sinibaldi L, Valenti P, Orsi N. Metal complexes of lactoferrin and their effect on the intracellular multiplication of *Legionella pneumophila.* Biometals. 2000;13:15–22. 10.1023/A:1009221616623 10831220

[37] James BW, Mauchline WS, Dennis PJ, Keevil CW. A study of iron acquisition mechanisms of *Legionella pneumophila* grown in chemostat culture. Curr Microbiol. 1997;34:238–43. 10.1007/s002849900176 9058545

[38] Kusnetsov J, Iivanainen E, Elomaa N, Zacheus O, Martikainen PJ. Copper and silver ions more effective against legionellae than against mycobacteria in a hospital warm water system. Water Res. 2001;35:4217–25. 10.1016/S0043-1354(01)00124-5 11791852

[39] Rogers J, Dowsett AB, Dennis PJ, Lee JV, Keevil CW. Influence of temperature and plumbing material selection on biofilm formation and growth of *Legionella pneumophila* in a model potable water system containing complex microbial flora. Appl Environ Microbiol. 1994;60:1585–92. 8017938 10.1128/aem.60.5.1585-1592.1994 PMC201521

[40] Liu Z, Stout JE, Boldin M, Rugh J, Diven WF, Yu VL. Intermittent use of copper-silver ionization for *Legionella* control in water distribution systems: a potential option in buildings housing individuals at low risk of infection. Clin Infect Dis. 1998;26:138–40. 10.1086/516283 9455522

[41] Hambidge A. Reviewing efficacy of alternative water treatment techniques. Health Estate. 2001;55:23–5. 11447890

[42] Rohr U, Weber S, Selenka F, Wilhelm M. Impact of silver and copper on the survival of amoebae and ciliated protozoa in vitro. Int J Hyg Environ Health. 2000;203:87–9. 10.1078/S1438-4639(04)70013-9 10956595

[43] Ezzeddine H, Van Ossel C, Delmee M, Wauters C. *Legionella* spp. in a hospital hot water system: effect of control measures. J Hosp Infect. 1989;13:121–31. 10.1016/0195-6701(89)90018-2 2567304

[44] Heudorf U, Hentschel W, Hoffmann M, Luck C, Schubert R. Legionellae in domestic warm water effects on the health of residents. Gesundheitswesen. 2001;63:326–34. 10.1055/s-2001-14215 11441676

[45] Stout JE, Yu VL, Yee YC, Vaccarello S, Diven W, Lee TC. *Legionella pneumophila* in residential water supplies: environmental surveillance with clinical assessment for Legionnaires’ disease. Epidemiol Infect. 1992;109:49–57. 1499672 PMC2272241

[46] Borella P, Montagna MT, Romano-Spica V, Stampi S, Stancanelli G, Triassi M, Relationship between mineral content of domestic hot water and microbial contamination. J Trace Elem Med Biol. 2003;17(Suppl):37–43. 14650627

