# Correction Vol. 10, No. 3

**DOI:** 10.3201/eid1007.C111007

**Published:** 2004-07

**Keywords:** errata, erratum, correction

In “Legionella Infection Risk from Domestic Hot Water,” by Paola Borella et al., errors occurred in the abstract. The seventh sentence should read as follows:

“Furthermore, zinc levels of <100 μg/L and copper levels of >50 μg/L appeared to be protective against *Legionella *colonization.”

The corrected article appears online at http://wwwnc.cdc.gov/eid/article/10/3/02-0707_article.htm.

We regret any confusion these errors may have caused.

